# New Potential Pharmacological Functions of Chinese Herbal Medicines via Regulation of Autophagy

**DOI:** 10.3390/molecules21030359

**Published:** 2016-03-17

**Authors:** Betty Yuen Kwan Law, Simon Wing Fai Mok, An Guo Wu, Christopher Wai Kei Lam, Margaret Xin Yi Yu, Vincent Kam Wai Wong

**Affiliations:** State Key Laboratory of Quality Research in Chinese Medicine, Macau University of Science and Technology, Macau, China; yklaw@must.edu.mo (B.Y.K.L.); smok55@hotmail.com (S.W.F.M.); wag1114@foxmail.com (A.G.W.); wklam@must.edu.mo (C.W.K.L.); yxyworld@aliyun.com (M.S.Y.Y.)

**Keywords:** autophagy, natural products, novel functions, Chinese herbal medicines

## Abstract

Autophagy is a universal catabolic cellular process for quality control of cytoplasm and maintenance of cellular homeostasis upon nutrient deprivation and environmental stimulus. It involves the lysosomal degradation of cellular components such as misfolded proteins or damaged organelles. Defects in autophagy are implicated in the pathogenesis of diseases including cancers, myopathy, neurodegenerations, infections and cardiovascular diseases. In the recent decade, traditional drugs with new clinical applications are not only commonly found in Western medicines, but also highlighted in Chinese herbal medicines (CHM). For instance, pharmacological studies have revealed that active components or fractions from Chaihu (*Radix bupleuri*), Hu Zhang (*Rhizoma polygoni cuspidati*), Donglingcao (*Rabdosia rubesens*), Hou po (*Cortex magnoliae officinalis*) and Chuan xiong (*Rhizoma chuanxiong*) modulate cancers, neurodegeneration and cardiovascular disease via autophagy. These findings shed light on the potential new applications and formulation of CHM decoctions via regulation of autophagy. This article reviews the roles of autophagy in the pharmacological actions of CHM and discusses their new potential clinical applications in various human diseases.

## 1. Introduction

Autophagy is the catabolic process in which eukaryotic cells engulf and lysosomally digest intracellular contents. The process maintains metabolic balance and cellular quality by removing dysfunctional cytoplasmic constituents and recycling basic molecular building blocks. Macroautophagy envelops and delivers the unfavoured intracellular contents to lytic compartment through the doubled-membraned autophagosome [1].

Autophagy-related proteins (Atg) are the main underpinning mechanistic component of macroautophagy. About 30 mammalian homologs of yeast Atg have been discovered. These proteins are responsible for the nucleation and elongation of the isolation membrane through protein complex formation [2,3]. Basal autophagy retains proper physiological functioning of cells through the control of cellular homeostasis, metabolic balance, and protein structural integrity. Additionally, macroautophagy is known as type II programmed cell death with its molecular regulation intricately interconnecting with the apoptotic mechanism [4,5,6,7]. Growing evidence also suggests involvement of macroautophagy in innate and adaptive immunity [8,9]. Therefore hampered autophagy is closely associated with proteinopathies, metabolic and immunological disorders. In fact, macroautophagy failure is the molecular culprit of aberrant proteins accumulation in neurodegenerative diseases such as prion, Alzheimer’s and Parkinson’s [10,11,12]. In metabolic diseases, altered macroautophagy is related to obesity, hyperglycemia, hypertriglyceridemia, and hypoalphalipoproteinemia [13,14,15,16]. A murine model with autophagy related gene (Atg7) conditionally knockout from pancreatic β-cells induced hyperglycemia as a result of reduced insulin synthesis and secretion [17]. In addition, aging is the chronic recession of metabolism and cellular homeostasis, the diminishing macroautophagy over time leads to aged-related physiological and pathological changes, and is reversely related to longevity [18,19,20]. Also, dampened macroautophagy-induced cellular toxicity promotes tumorigenesis. The progression of prostate, breast and ovarian cancers, and brain tumors including glioblastoma and myeloma are accelerated accompanying dysregulated Atg activities [21,22,23]. The macroautophagy inducer metformin has demonstrated cytotoxicity against colon cancer cells with mechanism independent of p53 signaling [24]. Saikosaponin-d, (*Z*)-3,4,5,4′-*trans*-tetramethoxy-stilbene, liensinine, isoliensinine, dauricine and cepharanthine can be used to treat drug-resistant and apoptosis-resistant cancers by triggering autophagic cell death [25,26,27]. Macroautophagy also regulates pathogens removal and immunocellular homeostasis maintenance suggesting its critical role in autoimmune and autoinflammatory disorders, for example, ulcerative colitis, Crohn’s disease, chronic obstructive pulmonary disease (COPD), systemic lupus erythematosus (SLE), rheumatoid arthritis (RA) and multiple sclerosis (MS) [28,29,30]. Clinical treatment with small molecules impeding macroautophagy activity, such as CQ (chloroquine), HCQ (hydroxychloroquine), FTY720 (a sphingosine analogue) and P140 peptide (a phosphopeptide), are therapeutically beneficial to lupus patients [31,32,33,34,35]. As such, macroautophagy has become an ideal pharmaceutical target for in-depth investigation. Up-to-date Chinese herbal medicine (CHM) researches have demonstrated that single small-molecules or herbal extracts isolated from herbal medicines such as *Bupleurum*, curcumin, *Polygonum cuspidatum*, *Rabdosia* rubescens, *Magnolia hypoleuca*, *Ligusticum wallichii franchat*, and *Panax notoginseng* can modulate macroautophagy with intervention effects towards tumorigenesis, infectious diseases, as well as neurodegenerative and cardiovascular impairments [36,37,38,39,40,41,42]. This review will discuss the context of macroautophagy (hereafter autophagy) regulatory mechanisms and their role in human diseases. Further discussion on specific CHMs will focus on their new therapeutic usage through regulation of autophagy.

## 2. The Molecular Circuitry Regulating Autophagy

### 2.1. The Ras-Raf1-MEK1/2-ERK1/2 Cascade

ERK activity is an autophagy inducing signaling cascade in the intestinal-derived cells effecting through the phosphorylation of GTPase-activating protein G α interacting protein (GAIP) by ERK which is phosphorylated by mitogen-activated protein kinase kinase (MEK1/2) [43]. The activated ERK also abrogates the autophagy inhibitory effects of the trimeric Gi3 protein [44]. Increased level of amino acid negatively regulates autophagy by phosphorylating the Ser^259^ of Raf1 (mitogen-activated protein kinase), the pivotal gatekeeper of the cascade [45].

### 2.2. The Beclin 1-Class III PtdIns3K Cascade

Amino acid starvation stimulates beclin 1-class III PtdIns3K interaction for protein complex formation through the assembly of beclin 1 with proteinaceous components like Vps34 (class III PtdIns3K) and p150 facilitating the upregulation of autophagy [46,47,48,49]. This process is independent of mTOR and is regulated by anti-apoptotic proteins which are regulated by JNK1- and death-associated protein kinase (DAPK) [50,51,52]. Nutrient deprivation activated DAPK to phosphorylate Thr^119^ within the BH3 domain which releases beclin 1 to bind with PtdIns3K for autophagy stimulation [51]. When subjected to starvation, JNK1 phosphorylate T^69^, S^70^, and S^87^ of Bcl-2 to detach beclin 1 from the beclin 1-BcL-2 complex for autophagy activation [50].

### 2.3. The mTOR-Mediated Cascade

The mammalian TOR performs its function by complexing with other proteins, and appears in two constitutively different forms which are the target of rapamycin complex 1 and 2 (mTORC1 and 2) [53]. Canonically, mTORC1 masters the repression of autophagy responding to growth factor, hormone and amino acid signaling by direct interaction with the Atg machinery and interference of autophagosome formation [54,55]. Compared with mTORC2, mTORC1 is better characterized which together with the TSC1/2 (tuberous sclerosis complex 1/2) complex, an mTORC1 activities suppressor, underpinning the central molecular governance of mTOR cascade.

#### 2.3.1. Class I Pxtdlns3K-Akt-mTORC1 Pathway

This pathway contributes mainly to the integration of growth factor and insulin signaling. The TSC1/2 complexes collect the signals from cellular sensor such as insulin receptor at the plasma membrane. Class I Ptdlns3K is then activated and phosphorylates phosphatidylinositol (4,5)-bisphosphate (PIP_2_) to phosphatidylinositol (3,4,5)-bisphosphate (PIP_3_). While class III PtdIns3K product PI3P is critical for autophagy, PIP_3_ generated by class I Ptdlns3K are inhibitory. PIP_3_ subsequently recruits and activates PKB [56].

#### 2.3.2. 5′ Adenosine Monophosphate-Activated Protein Kinase (AMPK)-Mediated Pathway

During cellular energy stress, the AMP: ATP ratio is increased because of intracellular ATP depletion [57,58] triggering the upstream activator of AMPK, liver kinase B1 (LKB1 kinase) [59]. The activated AMPK can interact directly with the regulatory-associated protein of mTOR (Raptor) subunit of mTORC1 inactivating the protein complex thereby upregulating autophagy [60]. Cytosolic calcium ion (Ca^2+^) concentration is another positive stimulation of AMPK-induced autophagy which is initiated by Ca^2+^/calmodulin-dependent kinase kinase β (CaMKKβ) and endoplasmic reticulum-localized Bcl-2 [61].

## 3. Role of Manipulating Autophagy in the Pathogenesis of Human Diseases

### 3.1. Defects Related to Aggregate-Prone Proteins

Accumulation of misfolded proteins is the common feature of the different neurodegenerative diseases [62]. The self-eating property of autophagy is cytoprotective which assists the clearance of the defective β-sheets enriched protein structure [63,64,65,66,67]. Clinically, the beclin 1 level of AD’s brain are found to be significantly depressed [68]. Transgenic mouse overexpressing beclin 1 could improve Parkinson’s disease (PD) progression by reducing α-synuclein aggregation through the enhancement of autophagy [69]. Mutant huntingtin (Htt) forms, the main source of neurotoxic activities of Huntington disease (HD), are sensitive to beclin 1 level as demonstrated by the increased accumulation of Htt upon beclin 1 deficiency [70]. A mouse model of AD and *in vitro* studies showed that rapamycin effectively induced amyloid-β (Aβ) clearance, and soothed the cognitive deficit [71]. Lithium inhibits inositol monophosphatase (IMPase) which reduces free inositol and IP_3_ levels promotes the removal of Htt of HD and α-synuclein via autophagy [72,73]. Repressing IP_3_ synthesis with the sodium salts of carbamazepine and valproate could induce similar therapeutic effects in the experimental models of HD [73,74,75]. Another mTOR-independent autophagy inducer, trehalose, is also relevant to Htt, α-synuclein and tau aggregations [76,77].

### 3.2. Metabolic Disorders

#### 3.2.1. Tumorigenesis

Most tumor suppressors and oncogenes are actually cellular metabolism regulators responsible for metabolic pathways including aerobic glycolysis, glutaminolysis and one-carbon metabolism [78]. The major cellular energy monitor AMPK, and hence the downstream autophagy up-regulated by the kinase, are closely related to cancer progression in response to metabolic stresses. AMPK activators such as 5-aminoimidazole-4-carboxamide riboside (AICAR) are strongly cytotoxic to the *in vitro* models of hepatic, gastric and prostate cancers [79,80,81]. Many other molecular messengers along the autophagy pathways are cancer-related. For example, the induction of the oncogenic mTORC1 attenuates autophagy and support cancer development [81,82,83,84]. Clinical trials with cytotoxic drugs rapamycin, temsirolimus and everolimus targeting mTORC1 to induce autophagy have been reported [85]. Therefore, cancer intervention with the use of autophagy inducers can trigger both autophagic cell death and physiological changes, such as cell cycle arrest, on cancer cells. This is of particular interest for most cancers and malignancies in regard to apoptotic resistance towards chemotherapies [86].

#### 3.2.2. Other Metabolic-Related Abnormalities

The systemic balance of glucose and fatty acid is frequently lost in patients enduring metabolic syndromes such as obesity and glucose intolerance. It is evident that the corresponding complex regulatory networks are linked up by autophagy [87]. Chronic consumption of diets with high nutrient abnormally activates mTORC1 [88]. The *de novo* hepatic lipogenesis can then be stimulated by mTORC1 and its substrate sterol regulatory element-binding protein 1c (SREBP-1c) during the obese state [89,90], which further dampens the expressions of the Atgs LC3, beclin1, Atg5 and Atg7 and thus the autophagic process [91]. While activating autophagy with pharmacological inducers seems to be an ideal method for improving metabolic disorders, caloric restriction appears to be the best strategy for controlling these disorders [92].

### 3.3. Immune Disorders

#### 3.3.1. Infections Control

Autophagy functions as an immune effector and directly delivers the intracellular microorganism or their components [9] into the lysosome through a specific form of selective autophagy called xenophagy [93,94]. Several medically important bacterial pathogens, such as *Mycobacterium tuberculosis* (Mtb), group A *Streptococcus*, *Salmonella*, *Shigella* and *Listeria* can be degraded by xenophagy. The Sindbis virus, herpes simplex virus and the parasite *Toxoplasma gondii* can also be eliminated by xenophagy [93,95,96].

Beyond the invading microbes, autophagy also targets the innate and adaptive immunity for preventing infections. Autophagy negatively regulates IL-1β and IL-18 expression through maintaining the quality of the intracellular milieu [97]. The overexpression of IL-1β and IL-18 of lipopolysaccharides (LPS)-stimulated macrophages with the loss of Atg16L1 further clarified the role of autophagy in inflammatory immune responses [98]. *In vitro* studies have verified the potential of some autophagic inducers in infection therapy. The hormonally active form of vitamin D1, 25D3, enhanced macrophage autophagy and prevented human immunodeficiency virus (HIV) replication [99]. Similarly, the small molecule PDK1 inhibitor AR12 cleared *Francisella tularensis* [100] and *Salmonella enterica* Serovar Typhimurium [100]. The therapeutic effects of the antibiotics cocktails containing isoniazid and pyrazinamide towards Mtb-infected host cells also corresponded to their autophagy-inducing properties [101].

#### 3.3.2. Autoimmune Diseases and Auto-Inflammation

Autoimmunity is the aberrant activation of immune systems towards self-antigens due to central intolerance [102]. Autophagy is essential for loading the MHC II compartment with intracellular antigens, a process involving the Atg-lysosome interaction [103], implicating the importance of autophagy in the determination of CD4^+^ T lymphocytes receptor repertoire. Also, the autoreactive thymocytes that have been programed in thymus can be eliminated by autophagy [104]. Besides, the autophagic activity is positively correlated with macrophagic expression of the proinflammatory cytokines TNF-α and IL-6 which may also contribute to SLE pathogenesis [105]. Glucocorticoids [106], anti-CD20 mAb (Rituximab) [107], bortezomib (Velcade) [108], cyclosporine [109], rapamycin (sirolimus) [110] and vitamin D3 [111] can induce autophagy for ameliorating the symptoms of SLE.

### 3.4. The Autophagy Regulatory Effects of Herbal Medicine and Their Novel Usage

#### 3.4.1. Herbal Medicine as the Ideal Source of Autophagy Modulators

**Figure 1:**
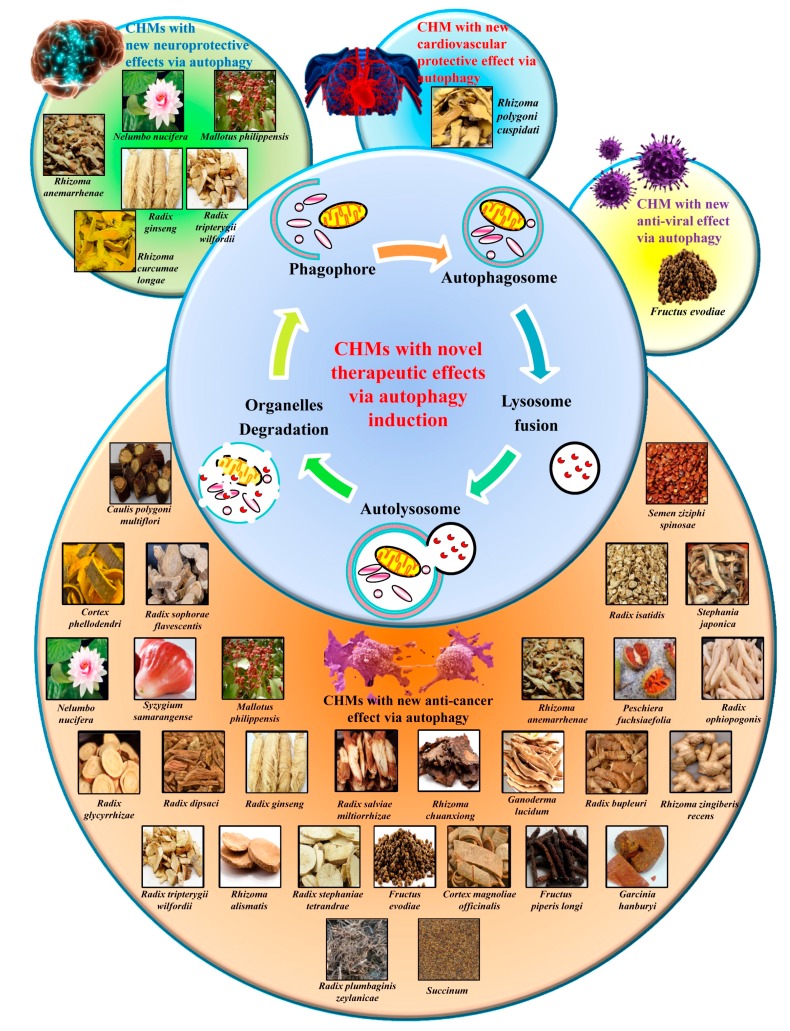
Novel therapeutic applications of CHMs via modulation of autophagy.

Since autophagy dysregulation underlies a broad range of pathological conditions, the successful therapeutic outcomes of using autophagy modulators, along with the expanding discoveries regarding the molecular basis of autophagy, suggest the need of intensively investigating the pharmaceutical potential of compounds with autophagy-adjusting ability (Table 1). While a number of synthetic autophagy regulators, such as tamoxifen, sorafenib and the water-soluble synthetic rapamycin temsirolimus, have been reported [112,113,114], natural autophagic compounds from CHMs are of interested because of their potential new therapeutic applications (Figure 1).

#### 3.4.2. Heat-Clearing Drugs

Heat-clearing drugs are used to clear damp-heat, fire or heat in the blood and body fluids to maintain regular body temperature and normal hemostatis of body [115,175].

*Radix scutellariae* (Huang qin) has been shown to modulate inflammatory diseases like gastroenteritis and hepatitis, and is also effective in controlling tumorigeneses [176]. The two main active components, baicalin and wogonin inhibit the release of proinflammatory mediators from different immunocellular components [177,178]. Baicalin and wogonin might be effective for inducing cytotoxicity or inhibiting proliferation in various human hepatoma [116]. The anti-cancer effects of *Radix scutellariae* such as induction of cell death and cell cycle arrest could be mediated through autophagy [179]. Although the involvement of autophagy in the *Radix scutellariae*-triggered anti-inflammatory property is still elusive, the progression of gastroenteritis and hepatitis are highly autophagic-related [180], suggesting the potential autophagic role of *Radix scutellariae* in such diseases. All these observations suggest that a main part of the clinical functions of *Radix scutellariae* as documented in Chinese traditional medical references are manifested via autophagy.

*Cortex phellodendri* (Huang bo) with berberine as its active ingredient after bark extraction has been traditionally prescribed for the treatment of pneumonia, tuberculosis, meningitis and liver cirrhosis [181,182]. *Cortex phellodendri* is anti-inflammatory in nature, which helps to eliminate invading pathogens, ameliorates acetaldehyde-induced hepatic NF-κB activation during cirrhosis [183,184], and inhibits glial proinflammatory iNOS (nitric oxide synthase) and TNF (tumor necrosis factor)-α activity [185]. Berberine modulates autophagic processes through the AMPK/mTOR signaling pathway [117,186], therefore, the observed anti-inflammatory capability of *Cortex phellodendri* was likely related to berberine-induced autophagy [118,187]. Recently, the dietary supplement Nexrutine^®^ which contains berberine, has been found to have therapeutic potential towards melanoma, multiple myeloma, prostate, pancreatic, breast and non-melanoma skin cancer [188,189,190,191,192]. Since berberine could induce both apoptosis and autophagy during tumorigenesis [193], autophagy may be responsible for the newly discovered anti-cancer properties of *Cortex phellodendri*.

*Rhizoma coptidis* (Huang lian), containing the active component berberine, has been traditionally used in alleviating inflammatory disorders, including type II diabetes. Pharmacological studies have reported the anti-inflammatory effects of *Rhizoma coptidis*. For example, berberine was effective in repressing the adverse responses caused by atherosclerosis (AS) [194]. *Rhizoma coptidis* was beneficial to the treatment of intestinal infections [195,196], and gut-associated abnormalities such as irritable bowel syndrome [197]. Furthermore, berberine induced autophagy [117,186] and suppressed the pro-inflammatory phenotype of macrophages [118]. In response to the anti-diabetic effect of *Rhizoma coptidis*, autophagy was important for regulating the synthesis and secretion of insulin by pancreatic β-cells [17]. Recent studies have also reported the potential of *Rhizoma coptidis* in neurodegeneration therapy [198,199]. With the protective role of autophagy in neurodegenative and inflammatory diseases, it is notable that the protective effect of *Rhizoma coptidis* may be regulated through autophagy.

*Radix sophorae flavescentis* (Ku shen) was used to alleviate toxicity, killed parasites and induced diuresis according to Chinese medicinal theory [200]. Many traditional formulas contain *Radix sophorae flavescentis*, for example, “Xiaofeng San” was prescribed for treating cutaneous disorders [201], and “Sanwu Huangqin Tang” has long been used for post-partum fevers arising from reproductive organ infections during childbirth [202]. These therapeutic effects suggest the immunomodulatory and anti-inflammatory function of *Radix sophorae flavescentis* may be attributed to the maintenance of systemic homeostasis by clearing off metabolic wastes through autophagy. The active component of *Radix sophorae flavescentis* is matrine, which is pharmacologically related to the suppression of inflammation by inhibiting neutrophil infiltration and oxidative stress, as well as reducing the production of inflammatory mediators [203]. Also, matrine has been reported as T cell anergy inducer through regulating the expression of anergy-associated genes such as Jumonji and CD98 [204]. The autophagic effects of matrine have suggested *Radix sophorae flavescentis* as a promising anti-cancer herb. For example, matrine was able to induce autophagic cell death against certain cancers [119,205,206], a pharmacological action which has not been reported in Chinese medicinal documents. Although no direct linkage between *Radix sophorae flavescentis*-induced autophagy and its traditional immunity regulatory effects has been reported, owing to the significant role of autophagy in immunomodulation, the involvement of such a process cannot be neglected.

*Herba rabdosiae* (Dong ling cao) has been extensively used in cancer therapy [207]. This herbal drug was also effective in encountering inflammation, oxidative stress and pathogen invasion [207]. Oridonin, an active component of *Herba rabdosiae*, attenuated neuroinflammation and associated oxidative stress by repressing the microglial production of nitric oxide and pro-inflammatory cytokines like IL-1b and IL-6 [208]. Oridonin inhibited cancer growth by repressing the expression of proinflammatory mediators like IL-33 and bone morphogenetic protein-2 (BMP-2) [209]. Besides, it was able to induce apoptosis and autophagic cell death in various cancer cell types, including esophageal cancer [120], prostate cancer [121], breast cancer [122], multiple myeloma [123], colorectal cancer [124], hepatoma carcinoma [125], and cervical carcinoma [126]. Therefore, the traditional anti-cancer property of *Herba rabdosiae* is highly correlated to autophagy.

*Radix isatidis* (Ban lan gen) is a popular herbal medicine, especially after the severe acute respiratory syndrome (SARS) epidemic, for its clinical applications in upper respiratory tract infections, including the nose, throat, and sinuses [210]. It is also prescribed for other viral infections like measles and hepatitis [211,212], *etc.* The active components of *Radix isatidis* are bis-benzylisoquinoline alkaloids—fangchinoline and tetrandrine. These active ingredients interact with invading pathogens and modulate the host responses. For example, fangchinoline could stop the replication of human immunodeficiency virus (HIV) Type 1 by disturbing the proteolytic process of viral gp160 [213]; tetrandrine suppressed hepatitis through the repression of NF-κB activation [214]. In addition, fangchinoline activated autophagic cell death through the p53/sestrin2/AMP pathway in hepatocellular carcinoma [127], whereas tetrandrine inhibited leukemia cell proliferation and induced autophagy *in vivo*. To our knowledge, the use of *Radix isatidis* in anti-cancer therapy has not been reported, therefore, these findings shed light to the development of the new usage of *Radix isatidis* in oncology through induction of autophagy. However, the role of autophagy in mediating the traditional anti-inflammatory function of *Radic isatidis* remains to be investigated.

*Stephania japonica* (Qian jin teng) has been traditionally used for relieving fever, diarrhea, dyspepsia and urinary disease [215]. Cepharanthine and dauricine are the active components of the herb that have been commonly used for inflammatory and immunological disorders in Chinese medicine [216,217]. Animal and *in vitro* studies illustrated that cepharanthine suppressed cytokine synthesis, platelet aggregation, plasma membrane lipid peroxidation, and nuclear factor-κB (NF-κB) stimulation [217]. The herb also facilitated the removal of free radicals and alleviated oxidative stress [218,219]. Dauricine exhibited similar anti-inflammatory effects through repressing the expression of inflammatory indicators such as myeloperoxidase, IL-1bβ and TNF-α [220]. Recently, these two compounds have been postulated as novel anti-cancer drugs. For example, cepharanthine abolished the drug resistance property of cancer cells by modulating the activities of multidrug resistance factor ABCC10 (MRP7) and ATPase [221]. Dauricine had been reported to suppress cancer proliferation and invasion, and promote apoptosis through the NF-κB signaling pathway [216]. Although documentation associating autophagy with the immunomodulatory effects of cepharanthine, dauricine or *Stephania japonica* is scarce, our laboratory discovered that both bioactive components induced autophagic cell death in apoptosis-resistant cancer cells [26]. These findings suggest cepharanthine and dauricine repress cancer growth through autophagy induction, and *Stephania japonica* may be a potential pharmaceutical agent for cancer.

*Rhizoma polygoni cuspidati* (Hu zhang) has been used to relieve inflammation, coughing, fever, and provide diuretic, emmenagogue, emollient and stomachic actions [222,223] and against tumor activity [223]. Resveratrol, the active compound of *Rhizoma polygoni cuspidati*, is a natural antibiotic [224] which inhibits the growth of bacteria and fungi [225,226]. Resveratrol exhibited its anti-inflammatory effect through inhibiting pro-inflammatory mediator synthesis [227]. Resveratrol also repressed tumorgenesis [228,229,230]. Increasing evidence has shown the autophagic role of resveratrol. For example, resveratrol attenuated the inflammatory phenotype of vascular endothelium through up-regulation of autophagy [128], and induced autophagic cell death in glioma and adenocarcinoma [129,130]. Recently, resveratrol-induced cardioprotection was found to associate with mTORC2-mediated autophagy and the up-regulation of antioxidant proteins [231,232]. Therefore, autophagy could be the underlying molecular mechanism responsible for the protective effects of *Rhizoma polygoni cuspidati*.

*Herba scutellariae barbatae* (Ban zhi lian) has long been used for cancer therapy [233]. It is also effective in treating inflammatory-related symptoms, for example, furunculosis, pyogenic infections, traumatic injury, edema, venomous snake bite, and jaundice [234]. The active ingredient of the herb, pheophorbide, inhibited cytokines expression and monocyte activities, down-regulated macrophage expression of iNOS, and scavenged reactive oxygen species (ROS) [235]. In addition, pheophorbide was cytotoxic to hepatocellular carcinoma [236] and breast adenocarcinoma [131]. Thus far, literatures correlating pheophorbide and autophagy were mostly about the photodynamic therapy of tumorigenesis involving the induction of autophagy and cancer cell death. In breast adenocarcinoma, pheophorbide induced autophagy through the ERK signaling pathway [131]. It also induced both apoptosis and autophagy via ERK1/2 and p38 in skin cancer [237]. Such autophagy-related therapeutic effect of pheophorbide has also been described in oral squamous carcinoma cells and hormone insensitive prostate cancer [132,133]. Accordingly, the anti-cancer effect of *Herba scutellariae barbatae* could be partly mediated by autophagy.

*Nelumbo nucifera* (Lian hua) has been used for maintaining homeostasis, reducing anxiety, acting against bleeding, and repressing inflammation [238]. Neferine is one of the active components responsible for maintaining glucose and lipid balance during starvation [239]. Anti-inflammatory and anti-oxidative effects of neferine in neurodegenerative disease by suppressing NF-κB activation and lipid peroxidation have also been reported [240]. Protective effects of neferine towards inflammation and oxidative stress were observed in pulmonary fibrosis [241]. Emerging findings suggest that autophagy is one of the pharmaceutical targets of *Nelumbo nucifera*. For example, neferine attenuated mutant huntingtin toxicity by inducing the AMPK-mTOR-dependent autophagic pathway [134]. Such findings correlated the psychopathological regulatory effects of neferine to autophagy. Neferine induced autophagy via the ROS mediated pathway in lung cancer [135], and was found effective in suppressing hepatocellular carcinoma [242]. These findings confirmed the role of the compound in controlling inflammatory progression and its novel usage in tumorigenesis. Therefore, triggering autophagy by *Nelumbo nucifera* could be a new pharmaceutical strategy for encountering inflammatory, neurodegenerative and cancerous diseases.

*Syzygium samarangense* (Lian wu) possessing the anti-free radical ability is indigenously used to manage inflammation-related conditions and removal of oxidative stress [243,244]. Dimethyl cardamonin (DMC) is the active compound isolated from the leaves of *Syzygium samarangense* contributing mainly to the anti-inflammatory and anti-oxidative properties. DMC protected the cells from ROS damage by modulating the glutathione S-transferase and superoxide dismutase (SOD) activities [245,246]. The compound also attenuated NF-κB activation and relieved cellular inflammatory phenotype with decreased serum level of proinflammatory cytokines [247,248]. Recent studies have suggested autophagy induction by DMC was associated with proliferative arrest in colorectal carcinoma by stimulating the p53/JNK-dependent signaling [249,250]. The therapeutic effects of DMC-induced autophagy have also been reported in cancers of the pancreas and prostate, myeloid leukemia and multiple myeloma [136]. Although the autophagic role of *Syzygium samarangense* in anti-inflammation remains to be elucidated, the induction of autophagy is highly correlated to the *Syzygium samarangense*-induced anti-cancer effects.

*Trichosanthes kirilowii* (Cucumber) has been used as folk medicine for the treatment of inflammation and cancer [251]. Pharmacological research further proposed *Trichosanthes kirilowii* as a potential remedy for suppressing HIV replication [252]. Cucurbitacin D, a major component of the herb, is an anti-inflammatory compound which inhibited pro-inflammatory mediator production via iNOS and NF-κB signaling [253,254] and cyclooxygenase-2 (COX-2) [255]. Cucurbitacin D was effective in the protection against hepatic and cardiovascular damages, the alleviation of diabetic condition, and the removal of invading microbes [256,257]. Cucurbitacin D could be used for repressing tumorigenesis of colorectal carcinoma [258], breast adenocarcinoma [259], and leukemia [260]. Recent findings have demonstrated that structural analogues of cucurbitacin D, for example, cucurbitacins B, E and I, induced autophagy and are potentially beneficial to cancer intervention. Cucurbitacin B triggered autophagy in breast cancer cells by manipulating the ROS activities [137], and in melanoma cells via c-Jun *N*-terminal kinase (JNK) activation [138]. Cucurbitacin E induced autophagy in cervical and breast cancer cells via activation of AMPK signaling [261]. Similar therapeutic effects have also been reported in cucurbitacin I treatment of glioblastoma [262]. All these observations point towards the likelihood of involvement of cucurbitacin D in autophagy-induced anti-tumor effects.

*Mallotus philippensis* (Cu kang chai) has been used for alleviating inflammatory symptoms caused by bronchitis, rheumatism and infection. The herb is useful in eliminating parasite invasions such as tapeworm [263]. Rottlerin, one of the major active components of the herb, also manifested potent anti-inflammatory properties. Rottlerin regulated inflammatory mediators including COX, protein kinase C δ, lipoxygenase, heme oxygenase and NF-κB [139]. Besides, it also suppressed the progression of malignant cancers by increasing the susceptibility of cancer cells towards apoptosis [264,265,266]. In addition, the anti-tumor effects of rottlerin were associated with its autophagy activation property in certain cell types [140,267,268]. Rottlerin induced apoptosis and autophagic cell death in prostate [141] and pancreatic cancers via the inhibition of PI3K/Akt/mTOR signaling [142]. Rottlerin also triggered apoptosis and autophagic cell death in fibrosarcoma cells through a PI3K/Akt/mTOR-independent pathway [140]. These findings, together with the protective role of rottlerin in preventing the spreading of misfolded proteins including prion protein, amyloid Aβ, and α-synuclein [139], suggest that *Mallotus philippensis* could be used as a novel therapeutic intervention for cancer-related and neurodegenerative disorders based on its autophagy-inducing ability. Whether autophagy is involved in the traditional anti-inflammatory effects of rottlerin or *Mallotus philippensis* is yet to be determined.

*Rhizoma anemarrhenae* (Zhi mu) has been used for minor symptoms like cough, fever and constipation [269,270]. The herb was also effective in treating diabetes [271]. One of the active components extracted from *Rhizoma anemarrhenae*, timosaponin AIII, relieved inflammation and oxidative damages by regulating the cytosolic Ca^2+^ concentration of endothelial cells [272], and neutrophilic superoxide generation stimulated by arachidonic acid [273]. Recent pharmacological studies demonstrated that the anti-tumor effect of timosaponin AIII was associated with autophagy. Timosaponin AIII elicited autophagy and cytotoxicity in cervical cancer which was independent of apoptosis [274]. It also repressed mTOR activity, triggered ER stress and autophagic cell death in breast cancer [275]. As demonstrated in an insulin resistance rodent model, timosaponin AIII-induced autophagy may be responsible for its diabetes-ameliorating effect through activation of AMPK [271]. In neurodegeneration model, timosaponin AIII activated autophagy and facilitated the downstream sequestration of aggregation-prone ubiquitinated proteins [143]. These findings implied the pharmaceutical potential of applying *Rhizoma anemarrhenae* for the treatment of cancers and neurodegenerative diseases.

#### 3.4.3. Tonifying Drugs

These herbs are used when the zheng qi (normal body condition or upright qi) is weakened, for example, during recovery from illness, during childhood or in old age. The herbs have the ability to tonify (*bu*), nourish, supplement and strengthen human body [115,276].

*Radix glycyrrhizae* (Liquorice, gan cao) is traditionally used as adjuvant to modify the efficacy of other herbs in a single prescription of around 80% of Chinese herbal formulas [277], which acted against inflammatory symptoms such as relieving cough, sore throat and phlegm production. It is important for maintaining a proper stomach function and is used for stomach ulcers. Licochalcone A (LA) and isoliquiritigenin (ISL) are compounds extracted from *Radix glycyrrhizae*. LA exhibited anti-inflammatory effects which suppressed pro-inflammatory mediator expression [278,279], and cleared cellular oxidative stress [280]. ISL demonstrated anti-oxidative [281] and immunomodulatory effects [282]. Of note, emerging data suggest that the anti-cancer properties in both compounds are associated with autophagy. LA induced autophagy via PI3K/Akt/mTOR signaling which repressed cervical cancer growth [283]. Androgen-sensitive prostate adenocarcinoma and adenoid cystic carcinoma were sensitive to LA- and ISL-induced autophagic cell death mediated by mTOR inhibition [146,284]. ISL also suppressed breast cancer progression through autophagy induction [285]. Therefore, *Radix glycyrrhizae* has the chemotherapeutic potential to function as an effective cancer therapeutic. Further investigation is needed for clarifying the mediating role of autophagy in the traditional anti-inflammatory effects of the herb.

*Radix dipsaci* (Xu duan) has been used for intervention in osteoporosis, strengthening of tendons and ligaments, and alleviating joint stiffness symptoms by promoting blood circulation in close proximity to the affected areas. The herb exhibits anti-inflammatory properties as reflected by its application in reducing abscesses, swellings, and sores [286]. In addition, *Radix dipsaci* helped to prevent abortion, which implies a regulatory role in the immune system [287]. Akebia saponin PA (AS) is one of the bioactive components found in *Radix dipsaci*, AS induced autophagic and apoptotic cell death of gastric cancer cells through both the AMPK/mTOR and PI3K/Akt/mTOR signaling and the downstream activation of p38/JNK molecular pathway, which facilitated capase-3-dependent apoptosis [147]. This finding pointed towards the potential therapeutic role of *Radix dipsaci* in cancers. Also, the possibility that autophagy may participate in the immunomodulatory and anti-inflammatory functions of *Radix dipsaci* should not be ignored.

*Radix ginseng* (Ren shen) has been prescribed for maintaining bioenergetics balance as suggested by Chinese herbalists for breathlessness, anorexia, hypodynamia, and diabetes [288]. Since, one of the main functions of autophagy is to retain energy homeostasis, it may be related to the traditional use of *ginseng* as mentioned. Pharmacological studies revealed that the bioactive ginsenosides including Rb1, Rg1, Rg3, Rh1, Re, and Rd [289] ameliorated inflammation, removed oxidative stress, stimulated immune system and regulated apoptosis. Therefore, *Radix ginseng* may cure more diseases than traditionally known. *Radix ginseng* illustrated beneficial effects in neurodegenerations in part due to its anti-oxidative [290] and anti-apoptotic properties [291]. Similar therapeutic uses of *Radix ginseng* have also been reported in cardiovascular disease [292], and cancers [293]. Recent researches have focused on studying the autophagic mechanisms of *Radix ginseng*. Rb1 suppressed neurotoxicity through inhibiting autophagy by beclin-1 downregulation [148]. The compound could also enhance cardiac muscle cell survival through autophagy [149]. The minor Rb1-derived ginsenoside F2 [294] and Rg3 could regulate autophagy leading to the suppression of breast cancer stem cells [295] and hepatocellular carcinoma [296], respectively, suggesting the role of autophagy in the potential new therapeutic action of ginseng.

*Peschiera fuchsiaefolia* (Dao zhong sha ma cha) showed *in vitro* antimalarial activity against *Plasmodium falciparum* [297]. Voacamine (VOA), a bioactive alkaloid extracted from the herb [298,299], has been reported to induce autophagic cell death of multidrug-resistant osteosarcoma, and inhibit the action of transporter P-glycoprotein (P-gp) [150]. VOA is the ligand of P-gp which expresses in kidney, gastrointestinal tract, brain, *etc.* [300]. In fact, diabetes mellitus is associated with P-gp dysregulation [301]. Also, the blood brain barrier (BBB) P-gp was associated with abnormal protein aggregation in Alzheimer's and Parkinson's diseases, suggesting the potential use of the herb in neurodegenerative disorders [302]. Therefore, the well-known autophagic involvement in diabetes mellitus and neurodegenerations strongly advocated that *Peschiera fuchsiaefolia* may act therapeutically as novel autophagy regulators under such pathological conditions.

*Radix ophiopogonis* (Mai dong) has been used for treating inflammatory symptoms such as cough and phlegm production, and cardiovascular diseases [303]. Ophiopogonin (OP)-B is one of the bioactive components, and was found to be an inducer of autophagy. In non-small cell lung cancer, OP-B up-regulated autophagy of tumor cells through PI3K/Akt pathways, and induced apoptosis-independent cell death and silences [144]. Another active constituent, OP-D exhibited anti-inflammatory effects through direct inhibition of mitochondrial ROS synthesis [145]. However, such an anti-inflammatory effect was not related to OP-D-induced upregulation of autophagy, as the compound could in fact suppress autophagy *per se* [145]. Therefore, owing to the close relationship between inflammation progression and autophagy, together with the autophagy modulating role of OP-B and OP-D, it is predicted that the compounds may contribute to the anti-inflammatory activity of the herb by regulating autophagy. Also, the findings of OP-B in inducing autophagic cancer cell death suggest the alternative use of *Radix ophiopogonis* in cancer therapy.

#### 3.4.4. Exterior-Releasing Drugs

These drugs help our body defenses against external stimulus, including invading pathogens, cold, heat, damp-wind or summer heat that may have a noxious effects on the human body, by maintaining a normal and healthy status of organ such as the stomach (wei qi) [115,276].

*Radix bupleuri* (Chai hu) has been used for counteracting different inflammatory conditions including pancreatitis, liver cirrhosis and fever, infections like malaria and common cold, modulating abnormal lipid metabolism and relieving depression [304]. The bioactivities of the main components, the saikosaponins, are responsible for the mentioned clinical indications. For instance, saikosaponins modulated host immunity by manipulating pro-inflammatory mediator release and lymphocyte responses [305]. Saikosaponins also repressed viral replication [306] and fever [307], reduced hepatotoxicity [308] and acted as tranquilizers [306]. Direct evidence for the participation of autophagy in regulating such activities is however lacking. However, contemporary studies verified the anti-tumor effect of saikosaponins, in particular Ssd (saikosaponin-d), through autophagy regulation. Ssd was cytotoxic to different cancers, such as breast and cervical cancers by increasing autophagy-induced ER stress via the CaMKKβ-AMPK-mTOR signaling [25,31]. In fact, formulated decoctions containing *Radix bupleuri* have been prescribed for cancer therapy [309], which supports the idea that *Radix bupleuri* is a novel autophagy enhancer exhibiting therapeutic effects towards cancers.

*Rhizoma zingiberis recens* (Sheng jiang) has been used for centuries for the treatment of colds, arthritis, migraines, nausea and hypertension [310,311]. 6-Gingerolis is the most abundant bioactive ingredient found in *Rhizoma zingiberis recens.* It suppressed oxidative stress by inhibiting iNOS activity of activated macrophage [312]. 6-Gingerol affected the anti-inflammatory properties by regulating Ca^2+^, which may be related to the regulation of autophagy [313]. The anti-mentic qualities of *Rhizoma zingiberis recens* are also related to 6-gingerol through inhibition of the function of serotonin 3 receptor [314]. In addition, *Rhizoma zingiberis recens*-mediated new anti-tumor functions via autophagy induction were further clarified. For instance, 6-gingerol induced cervical cancer cell death by upregulating caspase 3-mediated apoptosis, and autophagy partly via the repression of Akt signaling [315]. In pancreatic cancer, 6-gingerol induced cytotoxicity exclusively through autophagy by activating AMPK-mTOR signaling, which is a process independent of necroptosis and apoptosis [151].

#### 3.4.5. Wind-Dampness Dispelling Drugs

These herbs are responsible for treating painful obstruction (*bi*) syndromes due to wind (wind-*bi* syndrome), cold (cold-*bi* syndrome), dampness (dampness-*bi* syndrome) or heat (heat-*bi* syndrome), which are caused by poor qi or blood circulation [115,276].

*Radix tripterygii wilfordii* (Lei gong teng) has been used for the treatment of inflammation and overactive immune system, including rheumatoid arthritis, systemic lupus erythematosus, and dermatomyositis [316]. Celastrol is the bioactive component responsible for exhibiting the anti-inflammatory and anti-oxidative effects for alleviating autoimmune disorders such as chronic inflammation, neurodegenerative disease, and asthma [317]. Recent findings suggested the use of celastrol in cancer therapy as demonstrated by its inhibitory effects in different tumors [318]. Emerging data revealed that autophagy was one of the molecular machineries mediating these celastrol-induced therapeutic functions. By regulating the ROS/JNK signaling pathway, celastrol triggered autophagy and apoptosis which further repressed the proliferation of osteosarcoma cells in both animal and cellular models [152]. Beside, autophagy induction by celastrol through the inhibition of PI3K/Akt/mTOR signaling, have demonstrated therapeutic potential in inflammatory disorders such as Crohn’s disease, which implies that the traditional functions of *Radix tripterygii* wilfordii are mediated through the regulation of autophagy [319]. The compound also prevented neurodegeneration by inducing autophagy in affected neuronal cell death via the targeting JNK and PTEN-Akt/mTOR network [153].

*Radix stephaniae tetrandrae* (Fang ji) is used for the treatment of edema, anti-hypertension and analgesic, and was usually used as decoction such as “Fang Ji Huang Qi Tang” [320]. The main bioactive constituents of the herb are fangchinoline and tetrandrine. Both compounds are able to modulate cytokine expression and exhibit anti-inflammatory effects [321]. Fangchinoline reduced blood glucose levels, scavenged free radicals and reduced oxidative stress [322]. Tetrandrine exhibited anti-hypertensive action by disrupting Ca^2+^ movement and NO synthase activity [323]. Although the role of autophagy modulation in such alteration of glucose and Ca^2+^ level remains elusive, both fangchinoline and tetrandrine have been reported to contribute their anti-tumor effect through autophagy. The proliferation and invasion of gastric cancer cells could be suppressed by fangchinoline mediated inhibition of PI3K/AKT signaling [324]. The compound also triggered autophagic cell death by targeting the p53/sestrin2/AMPK signaling in hepatocellular carcinoma [127]. In leukemia cells, autophagy was induced by tetrandrine through the upregulation of Notch1 and ROS signaling [154]. These autophagy-related anti-tumor activity stimulated by fangchinoline and tetrandrine encourage the further development of *Radix stephaniae tetrandrae* as an effective drug for cancer therapy.

*Radix plumbaginis zeylanicae* (Bai hua dan) has effects in relieving pain, activating blood circulation, and is used for menstrual disorders, detoxification, and elimination of intestinal worms. Plumbagin, the bioactive component of the herb, is well known for its therapeutic safety and effectiveness [325]. Pharmacological studies demonstrated that plumbagin modulates the immune system and resolves inflammation [326,327]. The compound facilitates microbe clearance [328] and modulates lipid metabolism [329]. However, the role of autophagy in mediating these traditional functions of the herb remains to be elucidated. Recently, the anti-tumor activities of plumbagin have been increasingly reported [155,330]. The anti-tumor properties of plumbagin were attributed to the autophagy induction ability of the compound. For instance, plumbagin induced autophagic cell death in breast cancer via the AKT/mTOR signaling pathway [155]. The compound also triggered apoptosis and autophagic cell death in lung cancer and tongue squamous carcinoma cells through the mTOR signaling pathway [331].

#### 3.4.6. Dampness Draining and Transforming Drugs

These herbs are prescribed when there is an accumulation of dampness. This kind of disturbance in body fluid (water) metabolism is related to a dysfunction of the lung, spleen, kidney or bladder [115,276].

*Rhizoma alismatis* (Ze xie) is derived from the stem tuber of *Alisma orientale* [332]. The herb reduces the circulatory levels of cholesterol and blood sugar, and promotes urine production and perspiration, which helps to resolve symptoms like chronic nephritis and edema [333]. Alisol B is the active component of *Rhizoma alismatis* which improved lipid metabolism by inhibiting the absorption and synthesis of cholesterol [332]. Alisol B23-acetate, another important constituent of *Rhizoma alismatis*, participates in inhibiting antibody-mediated allergic reactions, and lipopolysaccharide (LPS)-induced iNOS and NO production [334]. Up to now, it is still questionable if autophagy mediates the traditional functions of *Rhizoma alismatis* or its bioactive constituents as mentioned. However, alisol B has been reported as a new autophagy inducer functioning through activation of CaMKK/AMPK/mTOR signaling, induction of apoptosis and triggering of cell death in breast cancer cells [156]. The anti-tumor effects of alisol B23-acetate have also been reported in hepatocellular carcinoma [156]. Therefore, *Rhizoma alismatis* has high potential to be developed as an anti-cancer drug through regulation of autophagy.

*Cortex magnoliae officinalis* (Hou Po) helps remove chest stuffiness due to phlegm accumulation, and relieves distension, which suggest its regulatory role in the immune system [335]. In consistence with the clinical indications of *Cortex magnoliae officinalis*, the bioactive component, magnolol, alleviated acute pain and endothelial damage stimulated by inflammation [336]. Other pharmacological effects of the compound included the reduction of anxiety and irritability via the regulation of γ-aminobutyric acid (GABA) receptor expression [337]. Magnolol also possessed anti-fungal properties [338], and was applied for bone repair by regulating the activities of osteoclasts and osteoblasts [157]. Magnolol also interplayed with the autophagic process which was beneficial to cancer therapy, but seemed not to be related to the traditional immunomodulatory effects of the herb. It induced autophagic cell death of lung cancer by blocking the PI3K/PTEN/Akt pathway [339]. Ery5, a compound derived from magnolol, activated autophagy and suppressed angiogenesis, causing apoptosis-independent cytotoxicity in prostate cancer cells [336]. Based on these observations, the effects of *Cortex magnoliae officinalis* on autophagy appear to be a valuable research niche in the search of new cancer treatment modalities.

#### 3.4.7. Interior Warming and Cold Expelling Drugs

These herbs are used to warm the interior organs, expel cold, tonify yang and rescue harmed yang qi and relieve pain. Interior cold of the human body can be caused by exogenous cold, or kidney yang deficiency which finally results in spleen and heart yang deficiency [115,276].

*Fructus evodiae* (Wu zhu yu) was effective for the treatment of gastrointestinal and menstrual disorders, postpartum hemorrhage and headaches [340,341]. Pharmacological studies of the bioactive component, evodiamine, showed that the compound is anti-inflammatory in nature by inhibiting COX-2 expression [342], and inducing blockage of preadipocytes differentiation [343]. Evodiamine also induced apoptosis and suppressed proliferation of cancer cells [158]. In addition, evodiamine could induce autophagic cell death in glioblastoma by quenching calcium/JNK signaling and apoptosis [158]. Through the modulation of beclin-1 and Bcl-2 expression, evodiamine induced autophagic cell death and apoptosis of gastric adenocarcinoma cells, respectively [344]. In a drug screening test, evodiamine was found to inhibit autophagic cell death of infected cells upon viral inoculation of influenza A through AMPK/TSC2/mTOR signaling [159]. Therefore, evodiamine regulated autophagy through a complex molecular network, further verification of such a circuit would help to discover and standardize the novel usage of evodiamine and *Fructus evodiae*. Further investigations correlating autophagy induced by evodiamine or *Fructus evodiae* to their traditional use should also be undertaken.

*Fructus piperis longi* (Bi bo) represses cough and fever, relieves allergic symptoms like asthma, helps cease pathogen invasions, reduces blood glucose level, induces coronary vasodilation and treats jaundice [345]. The active components, piperlongumine and its derivatives, inhibit pro-inflammatory mediator synthesis [346]. These compounds remove oxidative stress and prevent cardiac damage caused by ROS [347]. They also inhibit platelet aggregation and are used as crude drugs for promoting peripheral blood circulation [348]. Amongst the diverse pharmacological activities, the potential piperlongumine-induced cytotoxic effects towards the different cancer cells have aroused the most attention [349,350]. For example, autophagy induced by piperlongumine mediates the anti-tumor effects of the compound. Piperlongumine attenuates Akt/mTOR signaling and promotes autophagic cell death of cancer cells originated from breast, kidney, prostate and lung [160,161]. Piperlongumine induces autophagy by targeting the p38 signaling in osteosarcoma [351]. Apart from regulating tumorigenesis, the traditionally reported anti-inflammatory effect of the compound partly results from autophagy enhancement [352].

#### 3.4.8. Blood Regulating Drugs

These herbs are used to treat malfunctions in maintaining normal blood hemostasis, such as: (1) mild forms of blood stagnation caused by slow blood flow which may lead to blood stasis; (2) severe forms of stagnation due to congealment of phlegm, heat or cold, which lead to formation of solid masses and blood circulation stasis [115,276].

*Rhizoma curcumae longae* (Jiang huang) is a safe CHM for alleviating intermittent fever and inflammation of the bronchi, kidney and gall bladder, counteracting infections such as leprosy and cold, and treating of edema, diarrhea and cancer [353,354]. Curcumin, the main bioactive component of *Rhizoma curcumae longae*, possesses a unique structure amongst other active constituents extracted from the herb. Such characteristics conferred curcumin with pharmacological properties *per se* correlating with the clinical efficacy of *Rhizoma curcumae longae*. The compound has been associated with the capability of preventing inflammation [355], tumor progression [356], and oxidative stress accumulation [357]. Of note, curcumin was pharmacologically beneficial to neurodegenerative disorders [358,359]. The compound was particularly suitable for neurodegeneration intervention since the compound could cross the BBB after oral administration, acting as an anti-inflammatory and antioxidant drug, and amyloid aggregation inhibitor [360,361]. In a Parkinson’s disease model, curcumin triggered autophagy in neural cells by suppressing the mTOR/p70S6K signaling which hindered the downstream α-synuclein accumulation [162]. In addition, curcumin-induced autophagy was correlated to its anti-cancer properties. By the up-regulation of ERK1/2 and Akt/mTOR/p70S6K signaling, curcumin induced autophagy and suppressed the proliferation of glioma cells [163], suggesting the possible autophagic role of curcumin in various disease models.

*Radix salviae miltiorrhizae* (Dan shen) has been shown to prevent platelet aggregation and facilitate fibrinolysis [362]. It is a traditional remedy for managing coronary heart disease [363]. The herb was also used as ingredient in formulations for diabetes such as “tangzhiqing” [364], and was prescribed for hepatic and renal disorders [365]. Tanshinone IIA is the main bioactive component abundantly found in *Radix salviae miltiorrhizae*. It modulates inflammation and host immunity by acting on multiple targets depending on the cell types [366]. For instance, tanshinone IIA inhibited macrophagic NF-κB activation via the ERK1/2, p38 and JNK pathways [367]. In line with the clinical indications of *Radix salviae miltiorrhizae*, tanshinone IIA was cardioprotective through its action upon calcineurin/NFATc3 pathway [368]. Intriguingly, calcineurin regulated AMPK-dependent autophagy of cardiomyocytes upon oxidative stress [369] implying that the process may underlie the cardioprotective function of *Radix salviae miltiorrhizae*. In terms of autophagy regulation, tanshinone IIA has been reported to induce autophagic cell death of leukemia via activation of AMPK/mTOR and ERK/mTOR, as well as p70 S6K signaling [164]. Such observations suggested new potential uses of *Radix salviae miltiorrhizae* in the treatment of cancer via autophagy.

*Rhizoma chuanxiong* (Chuan xiong) is well known for its efficacy in improving blood fluidity, coronary and systemic circulation [370]. Contemporary studies have demonstrated that the herb modulates the proliferation of vascular smooth muscle cells [371] and prevents endothelial cell damage [372]. Ligustrazine (tetramethylpyrazine) is the active constituent which increases myocardial contractility and coronary circulation [373]. In addition, this single molecule acts as an anti-oxidant to remove superoxide anion, hydroxyl and lipid peroxyl radical which induce oxidative damages in tissues [374]. However, it is not known if autophagy is modulating the cardioprotective functions of the herb. Ligustrazine exhibits neuroprotective and anti-inflammatory effects on brain disorders such as cerebral ischemia, through elevating nuclear factor E2-related factor 2 (Nrf2)/heme oxygenase-1 (HO-1) expression [375]. Recently, ligustrazine has been reported to induce autophagy which was associated with cytotoxic effects toward hepatocellular carcinoma [37]. The ligustrazine-induced autophagic effect has also been demonstrated in protecting the kidney from neurotoxicity [165]. Therefore, the mechanisms underlying the *Rhizoma chuanxiong* or ligustrazine-induced autophagic process are intricate. Further investigations are needed to support the usage of the herb in pathological condition such as cancer and inflammation conditions.

#### 3.4.9. External Use Drugs

These kinds of compounds are applied to eliminate toxins, kill parasites, diminish swelling, relieve pain, expel pus and abscesses, improve wound healing, stop itching or bleeding [115,276].

*Venenum bufonis* (Chan su) is famous for its clinical application in resolving cardiovascular and inflammatory symptoms, including sore throat and tonsillitis, promoting urine production, and acting as an analgesic agent [376,377,378]. In modern Chinese medicine, *Venenum bufonis* was used in liver cancer therapy [378]. Bufalin is one of the constituents of *Venenum bufonis* exhibiting bioactivities related to the clinical indications described [379]. Recently, bufalin was found to regulate autophagy via cell type-dependent mechanisms to suppress tumorigenesis. In liver cancer, bufalin interacted with the Atg8, JNK, BECN-1 and TNF signaling, and stimulated autophagic cell death of hepatoma cells like Huh7, Hep3B and HA22T [380]. On the other hand, bufalin stopped the proliferation of hepatocellular carcinoma by activating autophagy through the Akt/mTOR and AMPK/mTOR pathways respectively [166]. The bufalin-induced autophagic cell death also effectively suppressed colon cancer cell proliferation via JNK activation [167], and the PTEN/AKT pathways [381]. Through targeting AMPK and the downstream p70S6K, bufalin modulated the apoptotic and autophagic activities of glioma cells [382]. Collectively, these findings support the traditional use of *Venenum bufonis* in the treatment of cancerous diseases via autophagy regulation.

*Garcinia hanburyi* (Teng huang) was used traditionally for treating inflammatory conditions and immunity dysregulation such as ulcerative gingivitis, skin infection, scald and burn, and chronic eczema [383]. The herb was also applied to cease traumatic bleeding, attacking toxin and parasites [383]. Gambogic acid is the major active ingredient of *Garcinia hanburyi* biologically alleviating pain, inflammation, and fever [384]. Recent investigations strongly suggest that gambogic acid is anti-tumor in nature by suppressing the proliferation of cancers of lung [385], liver [385], blood [386] and stomach [387]. Further studies revealed that gambogic acid triggered autophagy and ameliorated bladder cancer by modulating the beclin-1, p62 and NF-κB activities [168]. By up-regulating the beclin-1 expression, gambogic acid induced cytotoxic in leukemia cells through the induction of autophagy and apoptosis [169]. Apparently, *Garcinia hanburyi* and gambogic acid have the potential to be further developed as novel and effective anti-cancer therapeutic strategy. It is also important to verify if autophagy is mechanistically meditating the *Garcinia hanburyi*-induced inflammatory and immunological regulations.

#### 3.4.10. Spirit Calming Drugs

These herbs can be used to treat mental syndromes related to heart (blood and yin) deficiency such as over-activity of the heart, nervousness, fright, restlessness, irritability, insomnia, palpitations or anxiety; or treat syndromes related to liver (yin and yang) deficiency such as dizziness and headaches. This group of drugs is also prescribed to pacify internal wind which can contribute to tremor, spasms, paraesthesias of the limbs, dizziness or difficulties in walking [115,276].

*Radix polygalae* (RP) (Yuan zhi) is commonly prescribed in many classical decoctions such as “Kai Xin San” [388], and “Ding Zhi Wan” [389] for the treatment of forgetfulness [390], anxiety [391], insomnia or depression [392]. Recent pharmacological studies have also reported the sedative-hypnotic [391], memory improving [390], cognitive recognition enhancing [393], antidepressant [392] and neuroprotective effects [170] of RP. RP was reported to inhibit the phosphatidylinositol 3-kinase (PI3K)/Akt or activate the N-methyl-D-aspartate (NMDA) signaling pathways [394,395]. The active ingredients of RP such as onjisaponin B and other identified saponins, were proved to accelerate the clearance of neurodegenerative disease proteins such as huntingtin and α-synuclein, reduce aggregation and toxicity of mutant proteins through the induction of autophagy [170,396]. Therefore, RP may play its traditional sedative effect through degradation of unwanted proteins or organelles by autophagy.

*Ganoderma lucidum* (Ling zhi) is having efficacy in replenishing qi, stabilizing the nervous system and relieving cough and asthma, is commonly prescribed to treat insomnia, palpitation, cough and phlegm. It exerts tranquilizing effects through tonifying the heart, qi and blood. Triterpenes are the major components of *Ganoderma lucidum*. Pharmacological studies have demonstrated that both *Ganoderma lucidum* extract and ganoderic acid C2 could reduce accumulation of mutant huntingtins in PC-12 cells, alleviate neurotoxicity and behavioral deficits induced by 3-nitropropionic acid, and prevent or reverse memory loss resulting from sleep deprivation [171]. Additionally, it was also reported that *Ganoderma lucidum* triterpene extract (GLT) suppressed the proliferation of human colon cancer cells and inhibited tumor growth in a xenograft model, which were associated with the induction of autophagic cell death [397]. Furthermore, ganoderic acid activated autophagy, which facilitates immune recognition of CD4^+^ T cells; induced autophagic cell death and apoptosis of melanoma [398] *Ganoderma lucidum* triterpene extract induced autophagy which inhibited the development of colon cancer via p38 MAPK signaling [397]; and suppressed gastric cancer cells through the repression of p62 [399]. All these findings suggest that the traditional therapeutic role of *Ganoderma lucidum* is in part related to autophagy regulation, which may also be responsible for the novel use of the herb in cancer treatment.

*Caulis polygoni multiflori* (Shou wu teng) has been prescribed for nourishing blood, tranquilizing the mind and dispersing wind to treat insomnia, numbness of the skin and rheumatism in traditional Chinese medicine. It was often combined with *Semen ziziphi spinosae* (Suan zao ren) and *Cortex albiziae* (He huan pi) to treat insomnia, distraughtness and dizziness. Modern pharmacological study has suggested an effect of *Caulis polygoni multiflori* extract in protecting rats against CCl_4_-induced hepatotoxicity through its antioxidant activities [400]. The major active ingredients of Shou Wu Teng, anthraquinones, possessed similar chemical structures but different bioactivities. For example, emodin, the most abundant anthraquinone in rhubarb, inhibits cellular proliferation and prevents metastasis of cancers through apoptosis. Another major anthraquinone in rhubarb, rhein, inhibits the uptake of glucose and leads to cancer cell death caused by changes in membrane-associated functions [401]. Ananthraquinone-containing extract of Shou Wu Teng possesses myocardial protective effects by maintaining antioxidant status under oxidative stress conditions [402]. Abnormal aggregation of tau protein is highly correlated with the pathogenesis of Alzheimer’s disease (AD), therefore, with the ability in mitigating aggregation and cytotoxicity of tau [403] anthraquinones possesses high potential in modulating AD. Together with the fact that ananthraquinones were able to induce autophagic cell death in cancer cells [172], Shou Wu Teng may exert its traditional sedative function through the induction of autophagy, which was highly related to the modulation of neurodegenerative disease proteins, as well as cancers [172].

*Fructus schisandrae* (Wu wei zi) has been prescribed to replenish qi, nourish the kidneys and tranquilize the mind to produce a sedative nephroprotective effect. Besides, SC extract was co-administered with other medicine for reducing immunosuppressive drug (cyclosporine A)-induced side effects [404]. Schisandra total lignin (STL), the major active ingredient of *Fructus schisandrae*, delayed mouse brain aging by attenuating apoptosis [405]. *In vivo* experiments further demonstrated STL inhibited the d-galactose-induced brain tissue aging through regulating autophagy and inhibiting apoptosis in the mice. It has been reported that longevity-promoting regimens such as caloric restriction or inhibition of TOR is associated with induction of autophagy [173]. Therefore, autophagy may be the mechanism responsible for the sedative and anti-ageing effect of *Fructus schisandrae*.

*Semen ziziphi spinosae* (Suan zao ren) is commonly prescribed as “suan zao ren tang” for sedation, nourishing the nerves, insomnia, palpitations, anxiety, dizziness, dry mouth and throat, red tongue, and clinical treatment of neurasthenia, heart neurosis and menopausal syndrome due to deficiencies of the heart and liver [406,407]. The active component of *Semen ziziphi spinosae*, jujuboside B, was reported to inhibit platelet aggregation and target cardiovascular diseases associated with platelet hyperaggregation [408]. Furthermore, the anti-tumor activity of jujuboside B was reported to be associated with the induction of apoptosis and autophagy [409]. Another active neuroprotective component from *Semen ziziphi spinosae*, jujuboside A, could mitigate learning and memory impairment in mice, by reducing the level of Aβ_1–42_, and inhibiting the activities of acetylcholinesterase (AChE) and NO in the hippocampus and cerebral cortex of mice [410]. With its traditional effects in tranquilizing the mind and nourishing heart, blood and qi, current pharmacological studies have confirmed the neuroprotective and autophagic role of *Semen ziziphi spinosae*. As autophagy was highly correlated to the maintenance of cellular homeostasis, which was important for normal function of brain, autophagy may be responsible for the pharmacological action and sedative effects of *Semen ziziphi spinosae*.

*Succinum* (Ambrum) has been prescribed for relieving convulsion, tranquilizing the mind, activating blood and removing stasis, inducing diuresis, treating irritability, epilepsy, algomenorrhea and amenorrhea in TCM [411]. It was commonly prescribed with *Rhizoma acori graminei* (Shi Chang Pu) and *Radix polygalae* (Yuen Zhi) in “Hu Po Ding Zhi Wan” for treating palpitations, insomnia and forgetfulness [412]. Vitamin E succinate (VES), one of the active components of ambrum, was proved to induce autophagy via the inhibition of mTOR [174]. Besides, VES worked as an anti-neoplastic agent through regulating apoptosis in cancer cells [413]. As autophagy is also highly correlated with modulation of cancers, with the recently identified anti-cancer effect of jujuboside B and VES through induction of autophagy, both *Semen ziziphi spinosae* and Ambrum may possess new applications in anti-cancer therapy via its traditional tranquilizing effect, which was highly associated with the beneficial effect of autophagy. In addition, the Chinese medicinal herbs including *Nelumbo nucifera*, *Rhizoma acori graminei*, *Radix salviae miltiorrhizae* and *Radix ginseng* also participate in tranquilization of the mind [414]. *Nelumbo nucifera* treats palpitations, insomnia and dreamful sleep [415] in TCM. It is commonly prescribed as a formulation with *Radix polygalae*, *Semen ziziphi spinosae* and *Radix salviae miltiorrhizae*. The active component of *Nelumbo nucifera*, neferine, was identified as a novel autophagic enhancer which facilitates the degradation of mutant neurodegenerative disease proteins *in vitro* [134]. With the beneficial effect of autophagy in maintaining normal homeostasis in cells, sedative TCMs may play protective role in neurodegenerative diseases, through the removal of disease proteins by autophagy.

## 4. Current Clinical Application and Limitation in Applying Autophagic Modulators in Therapies

Preclinical or clinical models showed that pharmacological inhibition of autophagy can enhance the sensitivity of tumor cells towards multiple anti-cancer drugs. For example, inhibition of autophagy enhanced apoptosis induced by cetuximab [416] or vorinostat [417]. While CQ enhanced the therapeutic efficacy of saracatinib [418] in prostate cancer xenograft model, inhibition by 3-MA increased fluorouracil (5-FU) [419] induced apoptosis with tumor regression in colon cancer xenografts. Among these autophagy inhibitors, only CQ or HCQ were studied in humans as they cross the blood-brain barrier with HCQ much preferred to human due to the less severe side effects [420].

Based on these preclinical data, phase I or II trials were performed to evaluate the combinational use of autophagy inhibitors (HCQ or CQ) with various anti-cancer cytotoxic agents. However, there are limitations for their use in clinical practice due to the long half-life and high effective concentration of HCQ. A phase I trial was performed to evaluate the combined use of HCQ with temozolamide [421] and radiation in glioblastoma patients. Phase I or II clinical trials evaluating the combination use of bortezomib and CQ [422] are ongoing in patients with recurrent carcinoma. A phase I trial of 2-deoxyglucose [423], an agent that blocks glucose metabolism, showed a reduction in autophagy, suggesting the role of autophagy in cancer therapy.

Similarly, the clinically used mood stabilizers lithium (valproate and carbamazepine) [73], induce mTOR-independent autophagy and enhance the cellular degradation of aggregate-prone mutant huntingtin and α-synuclein. The hypertensive agent rilmenidine [424], a US Food and Drug Administration–approved compound, showed protective effect in Huntington’s disease models and is now under further clinical trials. Protein phosphatase 2A (PP2A) agonists [425] which favor induction of autophagy are currently under clinical trials for Alzheimer’s disease. The clinically used approved antidiabetic drug, metformin [426], attenuates disease development in some neurodegenerative diseases via AMPK activation.

2-Hydroxypropyl-β-cyclodextrin [427], effective in enhancing the clearance of lipids or autophagic substrate burden, can mitigate neurological deficits in an NPC mouse model and is currently under early clinical trials for treating NPC. Another lipofuscinolytic agent, centrophenoxine [428], is an anti-aging antioxidant which possesses potential protective effect for late-stage dementia in small early clinical trials. However, factors such as blood–brain barrier penetration power of the compounds and cellular heterogeneity of brain tissue can also affect the clinical neuroprotective efficacy of autophagy drugs. For example, the different responses of neurons and glia to autophagy or drug must be considered in clinical trial evaluations [429].

Although the molecular mechanisms and functions of many autophagy modulators isolated from CHM were intensively studied, we are still far away from translating these traditional herbs or compounds into clinical applications. Knowledge concerning autophagy research is mainly related to non-selective autophagy describing the molecular responses upon starvation. However, increasing studies are evidencing the significance of selective autophagy which involves specific molecular mediators targeting particular kinds of unwanted intracellular materials [430,431]. Therefore, it is important to clarify the potential discrepancies between selective and non-selective autophagy in terms of their response towards different therapeutic agents. On the other hand, the time of starting the autophagy modulatory treatment should also be considered. For example, early cancer development is likely to be prevented by autophagy induction, as cancer cells exploit nutrients generated by autophagy to survive under the stressful cellular environment at the later stages [432,433]. It is also important to take into account the cells type-specific property of autophagy which may otherwise minimize the efficacy of the applied herbs, and result in unfavourable side effects. The heterogeneous cell types as presented by hepatic lobules can well explain such a scenario. The liver consists mainly of parenchymal cells (hepatocytes) and around 40% of non-parenchymal cells such as hepatic stellate cells (HSCs) [434]. The functional consequences of autophagy induction on these cells vary under different diseased conditions. Autophagy of HSC activated during the end stage of chronic liver disease and hepatic fibrosis could be alleviated by autophagy repression *in vitro* [435,436,437]. In contrast, autophagy stimulation is beneficial upon most of the parenchymal cells-related hepatic malignancies. Therefore special caution would need to be taken when applying the autophagic modulators in cancer therapy.

## 5. Conclusions

An increasing number of Chinese herbal medicines (CHMs) have been discovered as autophagy modulators. Such autophagic-regulatory effects are therapeutically beneficial to a broad range of disorders which are consistent with their traditional usage. Interestingly, many CHM-induced autophagy findings described in this review also demonstrate novel applications in various pathological conditions. These potential therapeutic functions can be summarized into: (1) anti-cancer; (2) neuroprotective; (3) cardiovascular-protective; and (4) antiviral applications. For example, through the activation of autophagic cell death, tumorigeneses can be repressed by *Cortex phellodendri*, *Radix sophorae lavescentis*, *Radix isatidis*, and *Stephania japonica* which are not associated with cancer therapy according to Chinese herbology. In fact, a plethora of CHMs having similar potential in preventing cancer progression have not been studied before. These herbs include *Nelumbo nucifera*, *Syzygium samarangense*, *Mallotus philippensis*, *Rhizoma anemarrhenae*, *Radix glehniae*, *Radix ophiopogonis*, *Radix glycyrrhizae*, *Radix dipsaci*, *Radix ginseng*, *Radix codonopsis*, *Rhizoma atractylodis macrocephalae*, *Ganoderma lucidum*, *Radix bupleuri*, *Rhizoma zingiberis recens*, *Radix tripterygii wilfordii*, *Radix stephaniae tetrandrae*, *Rhizoma alismatis*, *Cortex magnoliae officinalis*, *Fructus piperis longi*, *Radix salviae miltiorrhizae*, *Rhizoma chuanxiong*, *Garcinia hanburyi*, *Radix plumbaginis zeylanicae*, and *Fructus evodiae*. In contrast, *Fructus evodiae* can inhibit autophagic cell death of influenza-infected cells which provides insight to the search of CHMs sharing such anti-viral properties. In the case of neurodegenerative disorders, *Nelumbo nucifera*, *Mallotus philippensis*, *Rhizoma anemarrhenae*, *Radix ginseng*, *Radix tripterygii wilfordii*, and *Rhizoma curcumae longae* can trigger previously unidentified autophagy-mediated neuronal survival. These groups of novel neuroprotective herbs may help remove misfolded protein aggregates via autophagy activation. Also, *Rhizoma polygoni cuspidate* can upregulate the cardiac myocytic autophagy to eliminate damaged proteins uncovering the innovative use of the herb in cardiovascular disorders. It should also be noted that a single CHM usually contain more than one bioactive component. Therefore, the downstream molecular networks modulated by these CHMs are complicated. Precise investigations concerning how CHMs may influence the intricate autophagy machinery is needed, and will be a major challenge for further developing CHM as practical autophagy regulators.

When compared with Western medicines, most of the reviewed autophagy modulators here are bioactive components constituting the CHMs, which have long been prescribed as decoctions or formulations in the Chinese community with well-known pharmacological action, toxicity or side effects. Therefore, clinical trials using these natural herbs may be more safe and reliable. Also, the philosophy of CHM emphasizes the comprehensive and persistent body balance, which tailors them to deal with the autophagy-related diseases, which are pathologically associated with the loss of overall cellular and physiological balance. In addition, the actions of CHM are usually multi-targeting [307,438], making them suitable medications for autophagy-related disorders which are usually polysymptomatic. Therefore, detailed and systematic investigation concerning the interaction between CHMs and autophagy-related disorders in a comprehensive molecular approach is needed. Positive findings in these areas could widen the scope of CHM applications by suggesting novel intervention strategies, which have not been mentioned in the traditional Chinese pharmacopeia.

## Figures and Tables

**Table 1:** Comparisons of the autophagic effects and traditional usages of CHMs and their active components.

Types of CHM	Name of CHM	Traditional Usage [115]	Active Component	Autophagic Effects
Heat-clearing drugs	*Radix scutellariae* (Huang qin)	Cools heat, drains fire, clears damp-heat, stops bleeding	Baicali, wogonin	Induction of autophagic cell death in SMMC-7721 cells [116]
	*Cortex phellodendri* (Huang bo)	Clears damp-heat and deficient heat, drains fire, detoxifies	Berberine	Alleviation of ox-LDL induced inflammatory factors by up-regulation of autophagy via AMPK/mTOR signaling pathway [117]
	*Rhizoma coptidis* (Huang lian)	Cools heat, drains fire, clears damp-heat, detoxifies	Berberine	Induction of autophagy and help suppressing the pro-inflammatory phenotype of macrophages [118]
	*Radix sophorae flavescentis* (Ku shen)	Clears damp-heat, stops itching, disinfects and detoxifies	Matrine	Induction of autophagic cell death against C6 glioma/SGC-7901/HepG2 cells [119]
	*Herba rabdosiae* (Dong ling cao)	Cools heat, detoxifies and disinfects, moves blood, relieves pain	Oridonin	Induction of autophagic cell death in cancer cells including esophageal, prostate, breast, colorectal, hepatoma carcinoma and cervical carcinoma [120,121,122,123,124,125,126]
	*Radix isatidis* (Ban lan gen)	Cools heat, disinfects and detoxifies, clears the throat, cools blood	Fangchinoline, tetrandrine	Fangchinoline activates autophagic cell death through the p53/sestrin2/ AMP pathway of hepatocellular carcinoma [127]
	*Stephania japonica* (Qian jin teng)	Cools heat and detoxifies, promotes urination	Cepharanthine, dauricine	Induction of autophagic effects in cancer cells including HeLa, A549, MCF-7, PC3, HepG2, Hep3B and H 1299 [26]
	*Rhizoma polygoni cuspidati* (Hu zhang)	Moves blood, relieves pain, expels damp-wind, cools heat	Resveratrol	Attenuation of the inflammatory phenotype of vascular endothelium and induction of autophagic cell death in glioma and adeno-carcinoma [128,129,130]
	*Herba scutellariae barbatae* (Ban zhi lian)	Cools heat, clears damp-heat, breaks up lumps, promotes urination	Pheophorbide	Induction of autophagic cell death in oral squamous carcinoma cells, hormone insensitive prostate cancer and breast adenocarcinoma [131,132,133]
	*Nelumbo nucifera* (Lian hua)	Drains summerheat, raises the yang, stops bleeding, cools heat	Neferine	Attenuation of mutant huntingtin toxicity in PC-12 cells and inhibition of A549 cell proliferation cells by inducing autophagy [134,135]
	*Syzygium samarangense* (Lian wu)	Clears heat, detoxifies, alleviates itching	Dimethyl cardamonin (DMC)	Induction of autophagic cell death in colorectal carcinoma, pancreas, prostate, myeloid leukemia and multiple myeloma cells [136]
	*Trichosanthes kirilowii* (Huang gua)	Clears heat and promotes urination, detoxifies	Cucurbitacin D, cucurbitacin B, E and I,	Induction of autophagy in breast cancer cells by activating the ROS and in melanoma via c-Jun *N*-terminal kinase (JNK) activation [137,138]
	*Mallotus philippensis* (Cu kang cha)	Clears heat and promotes urination	Rottlerin	Induction of cell death in fibrosarcoma, prostate and pancreatic cancer cells, and prevention of prion protein, amyloid Aβ and α-synuclein through autophagy [139,140,141,142]
	*Rhizoma anemarrhenae* (Zhi mu)	Clears heat, clears damp-heat, generates fluids, tonifies and supports the yin	Timosaponin AIII	Induction of autophagic cell death in breast cancer and facilitation of the downstream sequestration of aggregation-prone ubiquitinated proteins [143]
Tonifying Drugs	*Radix ophiopogon japonicas* (Mai dong)	Tonifies and nourishes the yin, generates fluids, clears the heart and calms the spirit, clears deficient heat	Ophiopogonin B (OP-B) Ophiopogonin D (OP D)	OP-B induces apoptosis-independent non-small cell lung cancer death and silences through autophagy [144]; OP-D inhibits autophagic activity partially accounting for its heart protective effects against DOX-induced toxicity [145]
	*Radix glycyrrhizae* (Gan cao)	Harmonizes and tonifies the qi, spleen and stomach, detoxifies	Licochalcone A, isoliquiritigenin	Induction of autophagic cell death in cervical, breast cancer, androgen-sensitive prostate adenocarcinoma and adenoid cystic carcinoma cancer cells [146]
	*Radix dipsaci* (Xu duan)	Tonifies yang and kidneys, strengthens sinews and bones	Akebia saponin	Induction of autophagic cell death in gastric cancer cell through both the AMPK/mTOR and PI3K/Akt/mTOR signaling pathways [147]
	*Radix ginseng* (Ren shen)	Harmonizes and tonifies the qi, raises the qi, generates fluids	Ginsenosides Rb1, Rg1, Rg3, Rh1, Re, and Rd	Rb1 suppresses neurotoxicity and breast cancer stem cells [148], Re enhances cardiac muscle cell survival through autophagy [149]
	*Peschiera fuchsiaefolia* (Dao zhong sha ma cha)	Harmonizes and tonifies the qi, spleen and stomach, dries damp	Voacamine	Induction of autophagic cell death of multidrug-resistant osteosarcoma, and inhibition of the action of transporter P-gp [150]
Exterior-releasing drugs	*Radix bupleuri* (Chai hu)	Releases the exterior, moves and regulates qi, raises qi and yang	Saikosaponins	Cytotoxic to breast and cervical cancers by increasing autophagy-induced ER stress via the CaMKKβ-AMPK-mTOR signaling [25]
	*Rhizoma zingiberis recens* (Sheng jiang)	Releases the exterior, dispels cold, transforms cold phlegm	6-gingerolis	Induction of autophagic cell death in cervical cancer cell partly via the repression of Akt signaling and in pancreatic cancer through activation of AMPK-mTOR signaling [151]
Wind-dampness dispelling drugs	*Radix tripterygii wilfordii* (Lei gong teng)	Cools heat, draws out toxins, reduces swelling and pain	Celastrol	Repression of the proliferation of osteosarcoma cells, preventing neurodegeneration, and ameliorating experimental colitis in IL-10 deficient mice through autophagy [152,153]
	*Radix stephaniae tetrandrae* (Fang ji)	Dispels wind-damp, relieves pain, disperses swelling	Fangchinoline, tetrandrine	Fangchinoline induces autophagic cell death in hepatocellular carcinoma [127]. Tetrandrine induces autophagic cell death in leukemia cells [154]
	*Radix plumbaginis zeylanicae* (Bai hua dan)	Dispels wind-damp, relieves pain, disperses swelling	Plumbagin	Induction of autophagic cell death in breast cancer, lung cancer and tongue squamous carcinoma cells through the mTOR signaling pathway [155]
Dampness draining and transforming drugs	*Rhizoma alismatis* (Ze xie)	Promotes urination, drains dampness, clears damp-heat, clears deficient fire	Alisol B, alisol B23-acetate	Induction of autophagic cell death through activation of the CaMKK/AMPK/mTOR signaling pathway [156]
	*Cortex magnoliae officinalis* (Hou po)	Transforms dampness, breaks up stagnation, moves and regulates the qi	Magnolol	Induction of autophagic cell death of lung cancer by blocking the PI3K/PTEN/Akt pathway [157]
Interior warming and cold expelling drugs	*Fructus evodiae* (Wu zhu yu)	Warms cold, disperses cold, relieves pain, directs qi downwards	Evodiamine	Induction of autophagic cells death in glioblastoma, gastric adenocarcinoma [158]; Inhibition of IAV-induced autophagic cell death [159]
	*Fructus piperis longi* (Bi bo)	Warms cold, expels cold, relieves pain	Piperlongumine	Promotion autophagic cell death of breast, kidney, prostate and lung cancer cells [160,161]
Blood regulating drugs	*Rhizoma curcumae longae* (Jiang huang)	Regulates blood, moves blood, moves and regulates qi, descends the qi	Curcumin	Hinders α-synuclein accumulation in neural cells and suppression of the proliferation of glioma cells through induction of autophagy [162,163]
	*Radix salviae miltiorrhizae* (Dan shen)	Moves blood, breaks up blood stasis, cools heat, cools blood	Tanshinone IIA	Induction of autophagic cell death of leukemia via activation of AMPK/mTOR, ERK/mTOR and p70 S6K signaling [164]
	*Ligusticum wallichii* (Chuan xiong)	Moves blood, moves and regulates qi, dispels wind	Ligustrazine	Induction of cytotoxic effects in hepatocellular carcinoma and protection of the kidney from neurotoxicity through autophagy [37,165]
External using drugs	*Venenum bufonis* (Chan su)	Opens the orifices, detoxifies, relieves pain	Bufalin	Induction of cell death in hepatoma cells and suppression of colon cancer cells proliferation through autophagy [166,167]
	*Gamboge* (Teng huang)	Detoxifies, disperses swelling, antiparasitic, alleviates itching	Gambogic acid	Amelioration of bladder cancer and induction of cytotoxic in leukemia cell through autophagy [168,169]
Spirit calming drugs	*Radix polygalae* (Yuan zhi)	Anchors the yang, dislodges phlegm, opens the orifices	Onjisaponin B	Acceleration of the degradation of mutant α-synuclein and huntingtin in PC-12 cells through autophagy [170]
	*Ganoderma lucidum* (Ling zhi)	Tonifies the heart and qi, Calms and anchors the spirit	Ganoderic acid C2	Reduction of accumulation of mutant huntingtins in PC-12 cells, Induction of autophagic cell death in melanoma cells [171]
	*Caulis polygoni multiflori* (Shou wu teng)	calms and anchors the spirit, anchors the yang	Anthraquinones	Induction of autophagic cell death in C6 and U251 [172]
	*Fructus schisandrae* (Wu wei zi)	Harmonizes and tonifies the yin and qi, secures the essence	Schisandra total lignin	Inhibition of D-galactose-induced brain tissue aging through autophagy [173]
	*Semen ziziphi spinosae* (Suan zao ren)	Tonifies yin and blood, astringes and collects, anchors the yang	Jujuboside A, jujuboside B	Jujuboside B induces autophagic cell death in AGS and HCT 116 human cancer cells and suppresses tumor growth [173]
	*Succinum* (Ambrum)	Calms and anchors the spirit, sedates and cools the heart	Vitamin E succinate (VES)	VES-induced autophagy participates in SGC-7901 cell protection by inhibiting mTOR axis phosphorylation [174]

## Abbreviations

The following abbreviations are used in this manuscript:

CHM	Chinese herbal medicine
CHMs	Chinese herbal medicines
COPD	Chronic obstructive pulmonary disease
SLE	Systemic lupus erythematosus
RA	Rheumatoid arthritis
MS	Multiple sclerosis
CQ	Chloroquine
HCQ	Hydroxychloroquine
GAIP	G α interacting protein
MEK1/2	Mitogen-activated protein kinase kinase
Vps34	Class III PtdIns3K
DAPK	Death-associated protein kinase
mTORC1 and 2	Target of rapamycin complexes 1 and 2
PIP_2_	Phosphorylates phosphatidylinositol (4,5)-bisphosphate
PIP_3_	Phosphatidylinositol (3,4,5)-bisphosphate
Liver kinase B1	LKB1 kinase
Htt	Huntingtin
ATG	Autophagy-related proteins
ROS	Reactive oxygen species
ERK	Extracellular signal-regulated kinases
TSC	Tuberous Sclerosis Complex
AD	Alzheimer’s Disease
PD	Parkinson’s Disease
HD	Huntington disease
IMPase	Inositol monophosphatase
AICAR	5-aminoimidazole-4-carboxamide riboside
SREBP-1c	Sterol regulatory element-binding protein 1c
Mtb	*Mycobacterium tuberculosis*
HIV	Human immunodeficiency virus
JNK	c-Jun *N*-terminal kinase
NOS	Nitric oxide synthase
TNF	Tumor necrosis factor
AS	Atherosclerosis
NF-κB	Nuclear factor-κB
BMP-2	Bone morphogenetic protein-2
SARS	Severe acute respiratory syndrome
SOD	Superoxide dismutase
COX-2	Cyclooxygenase-2
VOA	Voacamine
P-gp	P-glycoprotein
OP	Ophiopogonin
BBB	Blood brain barrier
Ssd	Saikosaponin-d
LPS	Lipopolysaccharide
GABA	γ-aminobutyric acid
Nrf2	Nuclear factor E2-related factor 2
HO-1	Heme oxygenase-1
NMDA	N-methyl-D-aspartate
PI3K	Phosphatidylinositol 3-kinase
GLT	*Ganoderma lucidum* triterpene extract
STL	Schisandra total lignin
AChE	Acetylcholinesterase
VES	Vitamin E succinate
5-FU	Fluorouracil
PP2A	Protein phosphatase 2A
HSCs	Hepatic stellate cells
LA	Licochalcone A
AMPK	5′ adenosine monophosphate-activated protein kinase
CaMKKβ	Ca^2+^/calmodulin-dependent kinase kinase β
mTOR	mammalian target of rapamycin
NPC	Nasopharyngeal carcinoma
RP	*Radix polygalae*
SNF1	Sucrose non-fermenting 1
p70 S6K	p70 ribosomal protein S6 kinase
